# Polymorphism in the flanking regions of the Pb*GP43 *gene from the human pathogen *Paracoccidioides brasiliensis*: search for protein binding sequences and poly(A) cleavage sites

**DOI:** 10.1186/1471-2180-9-277

**Published:** 2009-12-30

**Authors:** Antonio A Rocha, Flávia V Morais, Rosana Puccia

**Affiliations:** 1 Department of Microbiology, Immunology and Parasitology, Federal University of São Paulo (UNIFESP), 04023-062 São Paulo, SP, Brazil; 2 Laboratory of Molecular Genetics and Genomics, University of Vale do Paraíba (UNIVAP), SP, Brazil

## Abstract

**Background:**

*Paracoccidioides brasiliensis *is a thermo-dimorphic fungus that causes paracoccidiodomycosis (PCM). Glycoprotein gp43 is the fungal main diagnostic antigen, which can also protect against murine PCM and interact with extracellular matrix proteins. It is structurally related to glucanases, however not active, and whose expression varies considerably. We have presently studied polymorphisms in the Pb*GP43 *flanking regions to help understand such variations.

**Results:**

we tested the protein-binding capacity of oligonucleotides covering the Pb*GP43 *proximal 5' flanking region, including overlap and mutated probes. We used electrophoretic mobility shift assays and found DNA binding regions between positions -134 to -103 and -255 to -215. Only mutation at -230, characteristic of *P. brasiliensis *phylogenetic species PS2, altered binding affinity. Next, we cloned and sequenced the 5' intergenic region up to position -2,047 from *P. brasiliensis *Pb339 and observed that it is composed of three tandem repetitive regions of about 500 bp preceded upstream by 442 bp. Correspondent PCR fragments of about 2,000 bp were found in eight out of fourteen isolates; in PS2 samples they were 1,500-bp long due to the absence of one repetitive region, as detected in Pb3. We also compared fifty-six Pb*GP43 *3' UTR sequences from ten isolates and have not observed polymorphisms; however we detected two main poly(A) clusters (1,420 to 1,441 and 1,451 to 1,457) of multiple cleavage sites. In a single isolate we found one to seven sites.

**Conclusions:**

We observed that the amount of Pb*GP43 *transcripts accumulated in *P. brasiliensis *Pb339 grown in defined medium was about 1,000-fold higher than in Pb18 and 120-fold higher than in Pb3. We have described a series of features in the gene flanking regions and differences among isolates, including DNA-binding sequences, which might impact gene regulation. Little is known about regulatory sequences in thermo-dimorphic fungi. The peculiar structure of tandem repetitive fragments in the 5' intergenic region of Pb*GP43*, their characteristic sequences, besides the presence of multiple poly(A) cleavage sites in the 3' UTR will certainly guide future studies.

## Background

*Paracoccidioides brasiliensis *is a thermo-dimorphic pathogenic fungus. It causes paracoccidiodomycosis (PCM) in man, which is an endemic mycosis in Latin America that affects mostly the lungs, but can disseminate to other organs [1]. *P. brasiliensis *is multinucleated in both pathogenic yeast and infectious mycelial phases. Genetic transformation in the species has recently been optimized [2], however genetic manipulation is still in its infancy. It is now recognized that most *P. brasiliensis *isolates diversified into an S1 main species, which is genetically close to the PS3 group of Colombian isolates, while PS2 is composed of a few isolates that constitute a phylogenetically cryptic species [3].

Gp43 is the main diagnostic and prognostic antigen so far characterized in *P. brasiliensis *[4,5]. It is a secretory glycoprotein whose peptide structure bears antigenic properties that are peculiar to the species [6]. Therefore, it confers high levels of sensitivity and specificity for PCM patients' sera when used as antigen in diagnostic tests such as immunodiffusion and capture ELISA, as well as by antigen detection in biological fluids [7]. Antibody titers are directly proportional to the severity of active PCM; they are probably not protective in advanced stages of the disease, but experimental protocols in mice point to the immunotherapeutic potential of anti-gp43 monoclonal antibodies [8]. On the other hand, gp43 contains T cell epitopes that are protective to vaccinated mice [5]. The best studied T-cell epitope is 15 aminoacid-long P-10, which showed additive effect in the treatment of murine PCM when administered with anti-fungal agents [9]. In addition, gp43 has adhesive properties to extracellular matrix proteins that may help fungal dissemination [10,11].

The complete Pb*GP43 *ORF has originally been found in a cloned 3,800-bp *Eco*RI genomic region from the Pb339 (B-339) isolate. It comprises 1,329 bp that contain a unique 78-bp intron [12]. The *Eco*RI genomic fragment includes 326 bp from the Pb*GP43 *5' intergenic proximal region and about 500 bp of the 3' intergenic sequence, which is shared by a neighboring *RanBP *homologue. This gene encodes a nuclear Ran-binding protein in *Schizosaccharomyces pombe*, or importin 11 in *Aspergillus fumigatus*, that transports ribosomal proteins to the nucleus [13]. Pb*GP43 *and Pb*RanBP *are linked in twelve *P. brasiliensis *isolates, as observed by Feitosa et al. [14].

Our group has carried out original and detailed studies on sequence polymorphism in the Pb*GP43 *ORF [15] and 5' intergenic proximal region [16], which defined at least five genotypes [17]. When compared to a consensus sequence, the most polymorphic A genotype carries three substitutions in the 5' intergenic proximal region and up to fifteen informative sites in the ORF, mostly concentrated in exon 2. So far, the A genotype has been detected in all six PS2 isolates [3]. It is of note that Pb*GP43 *was the most polymorphic gene in the multilocus analysis performed by Matute et al. [3] in *P. brasiliensis*. Isolates Pb2, Pb3 and Pb4, which belong in PS2 group [3], evoked milder experimental PCM in B10. A mice than representative isolates from the main species S1, including Pb18 [16]. This isolate has been long used in experimental PCM due to its high virulence.

*P. brasiliensis *Pb339 has traditionally been employed in antigen preparation [18]. It secretes high amounts of gp43, however that is not a rule among isolates [19]. The amount of gp43 accumulated in the extracellular fluids of a single isolate also varies with incubation time, culture medium, fungal phase, as well as with multiple sub-culturing after animal passage. In yeast-phase Pb339, extracellular gp43 decreases through late-log and stationary phases [18,20], when the culture pH tends to be basic [21]. Expression regulation of gp43 is only beginning to be unrevealed. Previous data from our group suggested that Pb*GP43 *suffers transcriptional regulation, but we showed that modulation at protein and secretion levels might also happen [16]. Besides, transcriptional response of Pb3 isolate to heat shock differed from others belonging to *P. brasiliensis *S1 group, suggesting that differences in Pb*GP43 *transcriptional regulation are likely to occur among isolates [16]. On the other hand, we have recently mapped NIT2-like binding motifs in the Pb*GP43 *5' intergenic region and described transcription modulation with nitrogen primary sources. In that case, the degree of modulation was similar among different isolates [22].

Differences in gp43 expression could be related to differences in transcription regulation due to genetic polymorphisms in the Pb*GP43 *flanking regions. In the present work, we found protein binding sequences in the proximal Pb*GP43 *5' flanking fragment and studied the effect of substitution sites; we characterized an extended 5' intergenic region up to 2,047 bp from Pb339 in comparison with other isolates and recognized some peculiar sequence organization. In addition, we studied polymorphism in the 3' UTR and polyadenylation cleavage site of the Pb*GP43 *transcript. Accumulation of Pb*GP43 *transcripts was much higher in Pb339 than in Pb18 and Pb3, however they were similarly modulated with glucose. The differences we presently found in the Pb339 5' intergenic region might help understand the features involved in differences of Pb*GP43 *transcriptional regulation.

## Results

### Search for DNA binding regions in the proximal Pb*GP43* 5' flanking region

**Figure 1:**
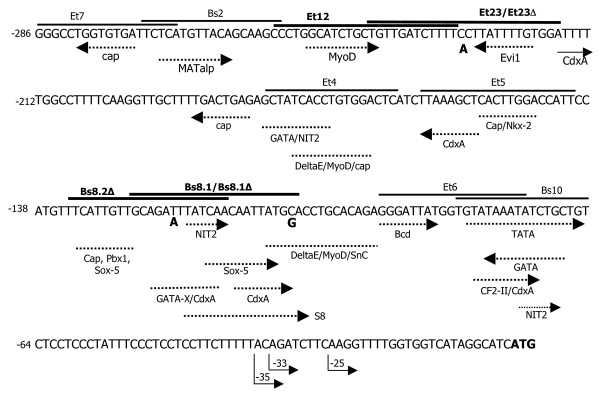
**Pb*GP43 *5' proximal flanking region from Pb339 between -286 and -1 showing the positions of oligonucleotides tested by EMSA and putative transcription motifs**. ATG start codon is bolded. Oligonucleotides that formed EMSA specific bands are indicated with bolded names. The three substitutions that occur in isolates from PS2 phylogenetic group are indicated at -104, -130, and -230, as well as the three transcription start sites mapped in four different isolates [16]. The positions of some putative transcription motifs detected with the TFsearch program http://www.cbrc.jp/research/db/TFSEARCH.html are indicated with the correspondent transcription factor. When the -120 (A) and -103 (G) mutations are considered, we point out disappearance of a GATA-X binding site and introduction of a C/EBP motif in Bs8.2Δ.

In order to find protein binding sites within the proximal 5' flanking region of the Pb*GP43 *gene cloned by Cisalpino et al. [12] we carried out EMSA using total protein extracts of *P. brasiliensis *and selected oligonucleotides (Table 1). Selection was based on the search for transcription factors using the TFSearch program (Figure 1) and DNAse I protection footprinting assays (data not shown), as established in our previous works [22,23]. We were aware of the incomplete type of information that transcription factor search programs could provide; however that was the strategy of choice to start our analysis. We were particularly interested to find DNA binding sequences in polymorphic regions.

**Table 1:** Sense oligonucleotides used in EMSA reactions

Et12	5' CCC TGG CAT CTG CTG TTG ATC TTT T 3'
Et23	5' CTG TTG ATC TTT TCC TTA TTT TGT GGA 3'
Et23Δ	5' CTG TTG ATC TTT TAC TTA TTT TGT GGA 3'
Et4	5' GCT ATC ACC TGT GGA CTC 3'
Et5	5' TTA AAG CTC ACT TGG ACC ATT 3'
Et6	5' GGG ATT ATG GTG TAT AAA TA 3'
Et7	5' AAG GGC CTG GTG TGA TTC TC 3'
Bs2	5' TTC TCA TGT TAC AGC A 3'
Bs8.1Δ	5' TGC AGA ATT ATC AAC AAT TAT GGA 3'
Bs8.1	5' TGC AGA TTT ATC AAC AAT TAT GCA 3'
Bs8.2Δ	5' TTC ATT GTT GCA GAA TTA TCA A 3'
Bs10	5' TGT ATA AAT ATC TGC TGT 3'

**Figure 2:**
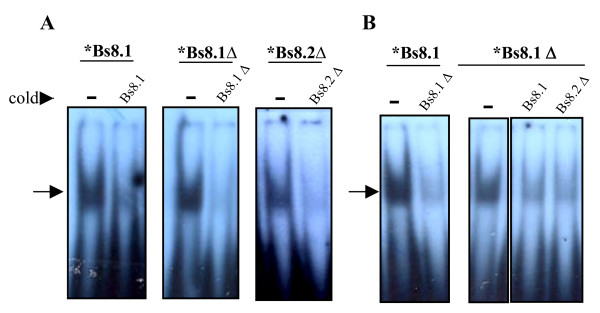
Radioautograms showing EMSA results with Pb339 protein extracts and radio labeled (*) Bs8.1, Bs8.1Δ, and Bs8.2Δ probes. In **A**, specificity of the EMSA bands was suggested by effective competition with 100 × molar excess of cold homologous probe. In **B**, cross-competition experiments with the indicated probes. Molar excess of cold competitors was 100 ×. The position of shifted bands is indicated with arrows.

The first set of probes tested by EMSA included Bs2, Bs8.1, Bs8.1Δ, Bs8.2Δ and Bs10 (Figure 1, Table 1). Bs2 and Bs10 resulted negative (data not shown), while the overlapping oligonucleotides Bs8.1, Bs8.1Δ and Bs8.2Δ (nt -134 to -103) formed intense shifted bands that were specifically inhibited with 100-fold excess of cold homologous probes, suggesting specificity (Figure 2A). Oligonucleotides Bs8.1Δ and Bs8.2Δ (nt -134 to -113) included substitutions at positions -120 (T/A) and/or -104 (C/G) that are characteristic of *P. brasiliensis *isolates belonging to phylogenetic species PS2, which is presently represented by Pb3 [3,15]. However, these substitutions did not seem to alter the intensity of protein binding (Figure 2A). In addition, probes Bs8.1, Bs8.1Δ and Bs8.2Δ cross-competed (Figure 2B). The Bs8.1, Bs8.1Δ and Bs8.2Δ complexes migrated similarly and the probes are similar in size (22 and 24 mer), suggesting binding to the same protein. Therefore, our results point to a protein binding core in the overlapping sequence TGCAGAA/TTTATCAA. Alternatively, all the probes are competing for distinct Sox-5-like protein binding sites (Figure 1). It is necessary to point out, however, that all the interpretations drawn from EMSA using total protein extracts will only possibly be confirmed by using either purified transcription factors or specific antibodies in super-shift experiments, considering that differences in shifts could be evoked by the same protein, while similar migrations could alternatively be the result of different transcription factors.

The next set of probes tested by EMSA included Et12, Et23, Et23Δ, Et4 and Et5 (Figure 1, Table 1). We tested these regions based on apparent protection in DNAse I protection footprinting assays (data not shown). In EMSA, probes Et4 and Et5 formed only weak and unspecific complexes with *P. brasiliensis *total protein extracts (data not shown), although these regions are rich in predicted transcription elements (Figure 1). We also tested an Et4 variant that had five extra upstream nucleotides. EMSA results were still negative, suggesting that the NIT2 motif predicted in this probe (Figure 1) is not functional.

**Figure 3:**
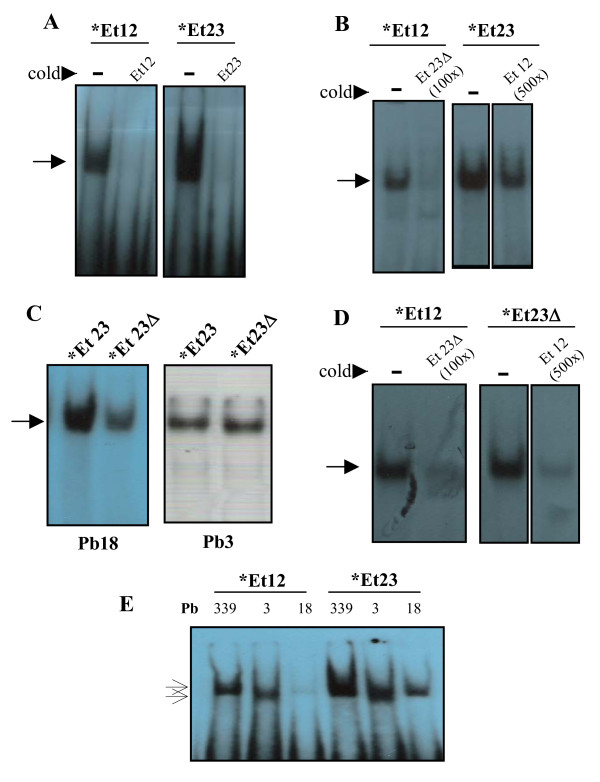
Radioautograms showing EMSA results with radio labeled (*) Et12, Et23, and Et23Δ probes. When not specified, protein extracts from Pb18 were used. In **A**, specificity of the EMSA bands was suggested by effective competition with 100 × molar excess of cold homologous probe. In **B and D**, cross-competition experiments with the indicated cold probes at 100 × or 500 × molar excess. In **C**, the intensity of Et23 and Et23Δ (mutated in -230 to A) bands are compared with different protein extracts (Pb3 or Pb18, as indicated). In **E**, migration of Et12 and Et23 bands are compared with protein extracts from different isolates (indicated). The position of shifted bands is indicated with arrows.

Overlapping Et12 and Et23 oligonucleotides (nt -255 to -215) formed intense complexes that co-migrated and could be specifically inhibited with 100-fold excess of cold homologous probe (Figure 3A). In cross-competition EMSA experiments, cold Et23 strongly competed with Et12, preventing band shift with as little as 100-fold molar excess. Cold Et12 was a weaker competitor to Et23 binding, since a noticeable decrease in band intensity demanded 500-fold molar excess of Et12 (Figure 3B). The results with Pb18 extracts presented in Figures 3A and 3B were similar with extracts from Pb339 and Pb3 (data not shown), suggesting that the same protein in each isolate binds to both probes; however affinity for Et23 is possibly higher. Therefore, a DNA binding motif might include the overlapping region from nt -243 to -229 (CTGTTGATCTTTT), for which there are no motifs recognized by the TFsearch computer program (Figure 1). We also designed an Et23Δ probe to verify the influence in EMSA of substitution at -230 (C/A). We initially noticed that the Et23Δ band was reproducibly less intense than the Et23 band when assayed with protein extracts from Pb18 (Figure 3C) and Pb339 (data not shown), but equally intense with Pb3 extracts (Figure 3C). In terms of competition with the Et12 complex, Et23Δ was as good a competitor as Et23, while cold Et12 could apparently inhibit band formation with Et23Δ more effectively than with Et23 (Figure 3D). Therefore, a C (instead of an A) at position -230 seems to be important for stronger Pb18 protein binding to Et23.

Figure 3E shows the Et12 and Et23 bands obtained with protein extracts from Pb18, Pb339 and Pb3 comparatively in the same radioautogram. It is noticeable that while the bands migrated similarly for each individual isolate, the Pb3 bands (both Et12 and Et23) migrated faster. It is worth mentioning that we observed similar behavior with Bs8.1Δ, which was also positive in EMSA with protein extracts from Pb18 and Pb3; the shifted band migrated similarly for Pb18 and Pb339, but faster for Pb3 (data not shown). Bs8.1 and Bs8.2Δ were only assayed with Pb339 extracts.

Manual search through the Pb*GP43 *promoter region revealed the existence of two CreA-like DNA binding motifs (C/GC/TGGA/GG), whose sequences (CTGGTG and ATGGTG) are observed in the Et6 and Et7 probes (Figure 1, Table 1). CreA is a zinc-finger catabolic repressor in *A. nidulans *[24] and we tested the probes with Pb339 extracts. We obtained an intense EMSA-positive band with Et7 that could only be removed with a 3,000-fold molar excess of cold oligonucleotide, suggesting lack of specificity (data not shown). Et6 formed only a faint band that disappeared upon competition with 250-fold molar excess of cold probe (data not shown).

### Analysis of 2,047 bp from the Pb*GP43* 5' flanking region

**Figure 4:**
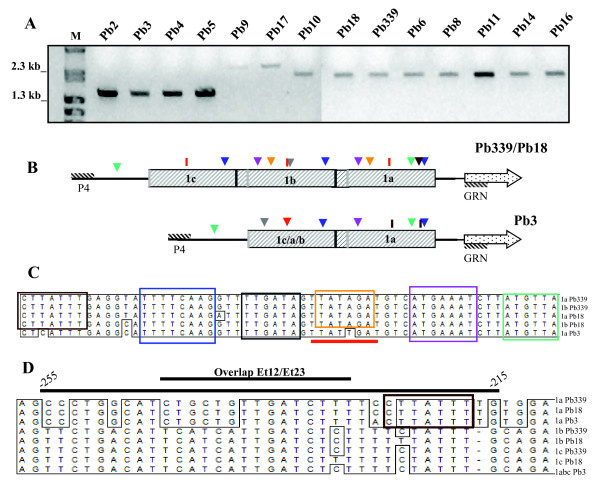
**Analysis of 2,047 bp upstream of the Pb*GP43 *ORF**. **A**, Size comparison of the Pb*GP43 *5' flanking region from fourteen *P. brasiliensis *isolates. Ethidium bromide-stained agarose gel showing the amplicons produced with P4 (forward) and GRN (reverse) primers using genomic DNA from the indicated isolates. M, molecular markers. **B**, schematic representation of the Pb*GP43 *5' flanking region from isolates Pb339/Pb18 and Pb3, where the positions of P4/GRN primers are shown. The repeated regions are boxed and start at the dark gray bar. The lighter-colored box indicates a 58-bp sequence ("connector", shown in **C**) that is absent in the upstream repeated region 1c and 1c/a/b. The sequences in the color-coded boxes can be found in the sites indicated in **B **by the correspondent colored arrow. **D**, sequence alignment of the Et12/Et23 probes (-255 to -215 in 1a region) with the correspondent fragments in other regions from Pb3, Pb18 and Pb339, as indicated. The overlap between these probes is indicated, as well as one of the connector sequences (brown) boxed in **C**.

In our laboratory, we had long been trying to clone an extended fragment of the 5' intergenic region of the Pb*GP43 *gene using different methods and Pb339 as reference isolate. Recently, we have finally managed to increase sequence information of this region to -2,047 bp (as detailed in Methods), which prompted us to search for length polymorphism in other isolates (Figures 4A). In order to do that, we compared PCR fragments amplified with P4 (forward) and GRN (reverse) primers (Figures 4B) and DNA template from 14 isolates (as coded in [15]). Note that amplicons from Pb2, Pb3, Pb4 and Pb5 had similar sizes of around 1,500 bp; amplicons from Pb9 and Pb17 were around 3,000 bp, while those from Pb6, Pb8, Pb10, Pb11, Pb14, Pb16 and Pb18 were similar to the original Pb339 fragment migrating at about 2,000 bp.

We next sequenced the Pb3 shorter PCR product; at a similar time frame the *P. brasiliensis *genome from isolates Pb3, Pb18 and Pb01 was released http://www.broad.mit.edu/annotation/genome/paracoccidioides_brasiliensis/MultiHome.html. Therefore, we had a chance to compare our sequences with those analyzed by the Broad Institute and the results are summarized in Figure 4.

We detected in Pb339 the presence of three consecutive repetitive regions: 1a (-652 to -156), 1b (-1159 to -653) and 1c (-1600 to -1158), which are about 500-bp long (Figure 4B). Two of the regions have initially been detected due to the difficulties to arrange the contigs generated through primer walking sequencing. A middle similar region has only been revealed very recently after further analysis of the data during preparation of this manuscript. The 1c region lacks an initial 58-bp fragment upstream (here referred to as "connector"), which is identical in 1a and 1b (Figure 4C). In Pb339, identities between regions are 89% (1a × 1b), 79% (1a × 1c) and 90% (1b × 1c). In Pb18, the structure and sequence of the Pb*GP43 *5' flanking region (2,047 bp) are quite similar to those in Pb339. Sequence identities are also high when comparing the same regions between Pb339 and Pb18: 99% (1a), 95% (1b) and 97% (1c). Pb3 lacks one repetitive region: 1a in Pb3 is 96% identical to 1a in Pb339, while 1c/a/b carries nucleotides characteristic of the three regions, however the level of identity is higher with 1c (94%) than with 1b (87%) or 1a (78%). Therefore, when sequence alignments of the repetitive regions from Pb339, Pb18 and Pb3 were compared in a dendrogram, there were two main clusters, one with 1a sequences and another branching into 1b and 1c (and 1c/a/b) regions (data not shown). Pb3 sequences formed individual branches, in accordance with the phylogenetically distinct nature of this isolate detected with Pb*GP43 *gene and other loci [3,15]. The 442-bp upstream fragment is highly divergent from the repetitive regions, but conserved among isolates (about 99% identity).

The highly conserved nature of the connector (Figure 4C) drove our attention to a more detailed analysis of its contents. We observed that some oligonucleotide sequences occur exclusively in the connectors, while others can be found in other positions of the repetitive regions. In Figure 4C, we boxed six sequences (6- to 8-bp long) that can be found in the positions represented in Figure 4B by color-coded arrowheads or bars. Note that the blue oligonucleotide (TTTTCAAG) was invariably found 44 bp upstream of the last base of all repetitive regions. The purple sequence (ATGAAAT) localized 109 bp downstream of the first base of the connector in the three isolates considered; therefore this sequence is not seen in 1c (or 1c/b/a) region. The gray sequence TTGATA in the connector could also be seen in 1b region at -883 (Pb339) and -1006 (Pb3). The green ATGTTA oligonucleotide was detected at -1756 (Pb339 and Pb18) and -1261 (Pb3) and at -268 in all isolates. The orange TATAGA was found exclusively in Pb18 and Pb339 at distances of 186 and 184 bp from the start base of 1a and 1b regions. The red-coded corresponding mutated sequence in Pb3 (TTATTGAT) was also detected 238 bp upstream of the last base in 1c/b/a region; it is not present in Pb18 or Pb339 connector, but it could be detected at distances varying among 237, 234 and 229 bp upstream of regions 1a, 1b and 1c last bases.

The brown CTTATTT initial connector sequence was observed only once in 1a region, 67 bp upstream of the last base in Pb339 and Pb18. Although this exact sequence is not observed in the Pb3 connector, which shows a unique CTTCATT oligonucleotide not found elsewhere, in this isolate CTTATTT has been observed twice in 1a region, at 67 bp upstream of the last base, and at a polymorphic -372 site. As seen in Figure 4D, CTTATTT is seen in protein-binding oligonucleotide Et23 (Figure 3), near the overlapped fragment with Et12. The Et12/23 fragment in 1a region is particularly polymorphic when compared to the correspondent sequences in 1b and 1c; it is one of the fingerprints of 1a region. In 1b and 1c regions, this fragment has several putative transcription motifs, as opposed to Et12/Et23 (Figure 1), however we have not tested their protein binding features.

### Polymorphism in the 3' UTR of Pb*GP43*

We compared the 3' UTR of the Pb*GP43 *gene by analyzing 3' RACE products from ten isolates. We used total RNA as template, which has been purified from *P. brasiliensis *yeast phase grown in rich medium (exception: Pb18, for which the mycelium phase was used). We sequenced the inserts of four to ten clones from each isolate and compared the poly(A) cleavage sites. In our hands, the 3' UTR was conserved intra and inter individuals, i.e., we have not found substitutions in all the 56 fragments sequenced (exception: site 1418 in a single clone from Pb14); however there was extensive polymorphism in the poly(A) cleavage site. Out of 56 transcripts we found thirteen close, however different poly(A) sites, which varied in number from one to seven per isolate (Table 2). These sites were located between positions 1420 and 1457 (91 to 128 nt from the stop codon, see inset in Table 2) and were mostly pyrimidineA, as precluded to occur in yeasts [25]. The most common sites were 1423 (14 transcripts) and 1434 (10 transcripts).

**Table 2:** Diversity in the Pb*GP43 *polyadenilation cleavage sites, which are also indicated (bold and italics) in the sequence below.

Cleavage sites	*P. brasiliensis *isolates											
	**1**	**2**	**3**	**4**	**5**	**7**	**8**	**10**	**12**	**14**	**clones/site**	**base**

**1420**						1					1	G

**1423**		4			5	2	1	2			14	C

**1425**				1							1	C*

**1427**			1					1	3	1	6	T

**1429**			1								1	C*

**1430**						1		1	1		3	T

**1434**	5			1			1		2	1	10	T

**1439**	1			1			2			1	5	G

**1441**						1		1	1		3	C

**1451**			1	1					1	1	4	C

**1453**			1	1							2	C*

**1454**					3				1		4	T

**1457**						1			1		2	T

**Total amplicons**	6	4	4	5	8	6	4	5	10	4	56	

1330tgggactttttacggcttggagcgtaggagaacagctgattatttacgtttacatgtttaacttttattaagaaatggaaaggcttaatt***g***aa***c***a***c***t***t***a***ct***aat***t***aatt***g***a***c***attgttttt***c***a***ct***ac***t***atccatttgtat 1470

* after this base there is a different base from A

Total RNA pools (isolated from cells cultivated in rich medium) used as template in the 3' RACE reactions were also analyzed for Pb*GP43 *expression using real time RT-PCR. The amount of accumulated transcript varied considerably among isolates (data not shown), from not detected (Pb2, Pb3, and Pb8) to highly abundant (Pb339, followed by Pb10) or low (Pb4, Pb12, Pb14, Pb18). There was no correlation between poly(A) cleavage site and Pb*GP43 *transcript accumulation in these experiments.

### Comparative analysis of Pb*GP43* transcription

**Figure 5:**
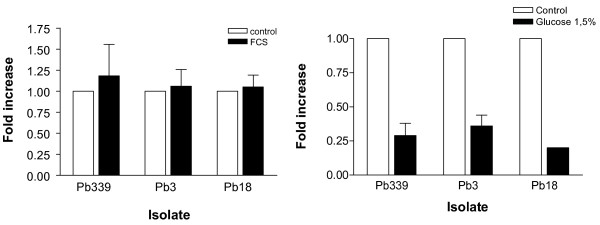
**Accumulation of Pb*GP43 *transcript after 30 min of stimulus of *P. brasiliensis *yeast cells with glucose or fetal calf serum (FCS)**. Real time RT-PCR experiments showing the relative variation of Pb*GP43 *transcript accumulation in Pb339, Pb18 and Pb3 cells stimulated with **A**, 2% FCS or **B**, 1,5% glucose. Control experiments were attributed value 1.0. The α-tubulin gene was used as standard.

Differences in extracellular gp43 expression among different isolates have been reported in the literature [19] and constantly observed in our laboratory. We have previously suggested that both transcriptional and post-transcriptional mechanisms would contribute to these differences [16]. Presently, we used Pb339, Pb3 and Pb18 in a controlled comparison of transcript accumulation in yeast cells cultivated to logarithmic phase in defined F12/glc medium. At similar cell concentrations for each culture, transcript accumulation was by far higher in Pb339, followed by Pb3 and Pb18 (Table 3). We have observed that differences were not apparent upon modulation with primary nitrogen sources, i.e., Pb*GP43 *transcript from Pb3, Pb18 and Pb339 were negatively modulated with ammonium sulfate at similar rates [22]. We presently tested two other types of stimuli in cultures growing in F12 medium, specifically, fetal calf serum (FCS) and glucose. As observed in Figure 5, supplementation with 2% FCS was not able to modulate Pb*GP43 *transcript accumulation in 30 min. On the other hand, an increase in glucose concentration from 0.18% (present in F12 medium) to 1.5% for 30 min evoked a decrease in the relative amount of transcripts of about 70% (2,6-fold for Pb3, 4-fold for Pb18 and 3,5-fold for Pb339). This rate of modulation was similar in Pb339, Pb3 and Pb18, although the initial amount of transcripts varied considerably among them. This kind of negative expression modulation with glucose would be expected for glucanase genes [26].

**Table 3:** Real time RT-PCR showing Pb*GP43 *transcript accumulation from three independent experiments, in which Pb339, Pb3 and Pb18 isolates were cultivated in F12/glc.

Isolate	Samples	TA	N° of cells/mL	N° of days
Pb339	Exp1	**3860 **± 51,5	9,2 × 10^6^	4
	Exp2	**4443 **± 25,6	1,1 × 10^7^	4
	Exp3	**10106 **± 108	1,6 × 10^7^	4
Pb3	Exp1	**41,6 **± 3,9	8,9 × 10^6^	4
	Exp2	**55,5 **± 4,3	1 × 10^7^	4
	Exp3	**51,66 **± 4,8	1,1 × 10^7^	4
Pb18	Exp1	**7,4 **± 0,8	1,4 × 10^7^	6
	Exp2	**4,1 **± 0,5	1 × 10^7^	6
	Exp3	**6,95 **± 0,5	1,2 × 10^7^	6

TA, relative number of transcript copies when compared with α-tubulin.

Culture densities and ages are indicated.

## Discussion

By using EMSA and a series of probes covering five regions within the upstream 326 bp of the Pb*GP43 *ORF we managed to identify protein binding sequences between nt -134 to -103 and nt -255 to -215. Together, these regions abrogate three substitution sites characteristic of *P. brasiliensis *PS2 isolates: that might not be incidental, since one mutation at -230 seemed to alter binding affinity. By using cross-competition assays with overlapping and mutated probes we suggested binding cores located between nt -125/-112 (TGCAGAA/TTTATCAA) and nt -243/-229 (CTGTTGATCTTTT). Fragment -125/-112 bears putative NIT2 and CdxA binding sites, whereas oligonucleotide from -243 to -229 could be involved in binding to a so far unknown protein. NIT2 modulates transcription of genes that encode enzymes involved in the catabolism of nitrogen sources during starvation [27]. We have recently studied Pb*GP43 *NIT2-binding sites and shown transcription modulation of the Pb*GP43 *with primary nitrogen sources; however the participation of a NIT2 transcription factor binding to the putative motifs at -179, -117 and -73 was unlikely [22]. The core sequence of CdxA-binding element is A/TA/TTA/TA/CTA/G [28], thus allowing for several sequence possibilities. That probably explains why CdxA is one of the most frequently found promoter elements in human genes [29]. Transcription factor CdxA is a homeodomain protein originally described in the early stages of morphogenesis of chicken intestinal tract [30], but its role on regulation of fungal genes has apparently not been addressed. The *P. brasiliensis *genome does not show any protein with high identities with CdxA, although other homeobox proteins have been annotated. On the other hand, there is a slight similarity of *P. brasiliensis *proteins with Sox-5, whose DNA-binding motif is seen in non-overlapping fragments of the probes covering sequence form -134 and -103.

To date, we have not been able to purify and identify the DNA-binding proteins detected here. The probes tested are located close to Pb*GP43 *transcription start sites and we understand from our previous work that the first -480 bp were sufficient to promote basal levels of gene transcription and also modulation with ammonium sulfate [22]. This fragment contains most of 1a region. When we blasted the overlap -125/-112 (14-mer) and -243/-229 (13-mer) oligonucleotides from EMSA-positive fragments with *P. brasiliensis *upstream intergenic regions http://www.broad.mit.edu/annotation/genome/paracoccidioides_brasiliensis/MultiHome.html, exact matches were found generally at the 11-mer level in about 20 to 30 genes. Sequence CTGTTGATCTTTT has been found in *P. brasiliensis *homologous genes encoding beta-hexosaminidase and chitin synthase, but mostly in genes encoding predicted or hypothetical proteins. Concerning the mutated -125/-112 region, we detected identical fragments in the upstream region of one gene encoding beta-glucosidase. Therefore, although gp43 is a non-functional β-1,3-exoglucanase, its gene may have conserved transcription motifs characteristic of those related to carbohydrate metabolism, possibly within the binding sequences identified here. We presently showed negative modulation with glucose of Pb*GP43 *from Pb3, Pb18 and Pb339 at similar rates, but the participation of the binding DNA sequences revealed here in this or other modulations is presently unknown and will have to be addressed using gene reporter experiments.

Basal levels of Pb*GP43 *transcripts varied considerably among isolates (Table 3) corroborating differences observed at extracellular protein levels [19]. By comparing length polymorphism of Pb*GP43 *upstream sequences we observed some correlation with *P. brasiliensis *phylogenetic group PS2 isolates, since DNA from Pb2, Pb3 and Pb4 yielded a similarly shorter amplicon of about 1,500 bp. However amplicon from Pb5 (S1 group [3] and Pb*GP43 *genotype D [17]) was also about this size. *P. brasiliensis *isolates representative of S1 group and Pb*GP43 *genotypes C, D, and E [17] resulted in amplification of a 2,000 bp-fragment, but exceptions of longer fragments were observed in Pb9 and Pb17 (S1, genotype E). It is possible that these isolates bear a forth repetitive region. We noticed that although the accumulated Pb*GP43 *transcripts in Pb339 can be as high as about 1,000-fold that of Pb18 (Table 2), this difference can not be justified by missing sequences within -2,047 to -1. In addition, even though there is one region missing in Pb3, accumulated Pb*GP43 *transcripts were only 129-fold less abundant than in Pb339. Therefore, the relevance of repetitive regions will be better investigated at the level of polymorphisms to explain transcription differences; however the influence of mRNA stability and 3' regulators should not be disregarded. Additionally, differences at the level of RNA processing should be better investigated. Several studies point to intraspecies divergence in gene expression related to mutations in *cis*-regulatory elements, such as in *Cyp6g 1 *(the cytochrome P450 family) from *Drosophila melanogaster *[31]. Changes in *cis*-regulatory systems of genes more often underlie the evolution of morphological diversity than do changes in gene number or protein function [32]. *Cis*-regulatory sequences are more susceptible to mutations; therefore long intergenic regions should accumulate them during evolution. It was surprising, however, to find highly conserved sequences among isolates upstream of the repetitive regions in the 5' intergenic region of Pb*GP43*.

We believe that the quite special arrangements detected in the 5' intergenic region of Pb*GP43 *are not at all incidental, however we can not precise their role at present. In addition, when we blasted the whole Pb339 connector sequence (58 bp) against other dimorphic fungal sequences http://www.broad.mit.edu/annotation/genome/dimorph_collab.1/MultiHome.html we realized that fragments of fifteen to thirteen bp or even longer (17 bp) are conserved in the 5' upstream regions from other genes, although mostly from predicted or hypothetical proteins. This specific search resulted in, for e.g., six matches with sequences from Pb18, three from Pb3, thirty-three from Pb01 and 13 from *H. capsulatum*. The sequence TTCAAGGTTTTGATAGTTATAG, including the blue and gray fragments (Figure 4C) was detected in the uracil DNA glycosidase superfamily from *H. capsulatum *H143; TATTTGAGGTATTTTCAA, including the brown and blue fragments (Figure 4C) was seen upstream of the acid phosphatase gene in the same isolate and part of it (ATTTGAGGTATTTT) was also found upstream of a reduced viability upon starvation protein in Pb18. Another fragment containing the red and pink sequences (Figure 4C) (TTATAGATGTCATGAAAT) is upstream of the MAP kinase gene in *H. capsulatum *H88.

Isolate Pb01 probably belongs to a different *Paracoccidioides *species whose proposed name is *P. lutzii *[33,34]. In this isolate, the gene homologue to Pb*GP43 *shows extensive polymorphism in the ORF, bearing only 80% identity with gp43 from Pb18. The predicted protein (PAAG 05770.1) does not have any *N*-glycosylation site, mutated NEP, or conserved P10, therefore it is a potentially active glucanase. The 5' intergenic region is reduced to about 990 bp, when the first exon from a gene homologous to that encoding succinate-semialdehyde dehydrogenase starts. In this fragment, we could observe one region that aligns with 1a, 1b and 1c regions, however with many divergences and two long gaps. Therefore, the transcripts are probably regulated differently, but there are no experimental data available to confirm that.

Protein binding probes were positive in EMSA carried out with total protein extracts from Pb339, Pb18 and Pb3; however EMSA bands migrated generally faster with Pb3 extracts and that could be related to the genetic differences found in isolates belonging to PS2. Interestingly, we observed that probes containing an AP-1 recognition sequence or heat shock elements within the shared 5' intergenic region between Pb*LON *and Pb*MDJ1 *formed EMSA bands that migrated consistently faster with protein extracts from Pb3 [23]. By comparing Pb3 and Pb18 AP-1 and HSF genome sequences, however, we observed that they are quite conserved; therefore polymorphism could not explain migration differences, which might be due to post-translational modifications in the translation factors or even binding to distinct proteins in different isolates.

One of the processing steps of pre-messenger RNA before export to the cytoplasm for translation involves endonucleolytic 3' cleavage for definition of the UTR and addition of the poly(A) tail. In higher eukaryotes, the choice of poly(A) sites involves, among others, a poly(A) signal (PAS) hexamer AAUAAA (or variants), localized 10 to 30 nt upstream of the poly(A) site, and U(U/G)-rich region (DSE) that lays 20 to 40 nt downstream of the poly(A) site [27,35]. The PAS hexamer binds to a poly(A) specific factor, while DSE bears binding sites to a cleavage stimulating factor that directs polyadenylation. In our studies we found multiple poly(A) cleavage sites between positions 1,420 and 1,457 of the Pb*GP43 *3' UTR. There is an AAGAAA sequence 21 nt upstream of position 1,420, which is a potential PAS, or positioning element as defined in yeast [25]. According to a survey on PAS hexamers in 13,942 human and 11,150 mouse genes [36], AAGAAA was the fifth most frequent PAS hexamer found, at a frequency of 2.99% in humans and 2.15% in mice. In the same study, the authors observed that heterogeneity of poly(A) cleavage sites occurred most frequently within a range of 24 bp. Therefore, they have defined poly(A) sites up to 24-bp long. They also noticed that the occurrence of multiple potentially alternative poly(A) sites in 54% of human genes. We analyzed 56 3' UTR sequences from a single gene (Pb*GP43*) in ten isolates of *P. brasiliensis *and observed that within a range of 37 bp there were two main clusters (1,420 to 1,441 and 1,451 to 1,457) of multiple cleavage sites separated by one to five bp (Table 2). They are separated by ten bp and we could speculate that they constitute two alternative poly(A) sites. Only 21% of the sequences (from six isolates) had cleavage sites in the second cluster. It is worth mentioning that the sequence downstream of the 3'-most cleavage site is U/UG-rich, as in mammal DSE, although this element has not been described in yeasts [25]. Differences in the PAS hexamer could result in diversity of cleavage sites. Our analysis showed conserved 3' UTR in the Pb*GP43*, therefore polymorphism in poly(A) cleavage site has a different origin. In yeasts, the role of close but alternative poly(A) site is unknown [25] and in *P. brasiliensis *this subject has originally been studied here. Comparison of 326 bp of Pb*GP43 *3' intergenic region from Pb339 (U2616.2) with genome sequences http://www.broad.mit.edu/annotation/genome/paracoccidioides_brasiliensis/MultiHome.html shows substitutions in positions 1,364, 1,385, 1,446, 1,563, 1,594 for Pb3 and Pb18, which have not been detected in the present work.

## Conclusions

We have undertaken extensive studies on polymorphisms in the 5' and 3' intergenic regions of the Pb*GP43 *gene from *Paracoccidioides brasiliensis*. We have characterized 2,047 bp of intergenic region and described a peculiar type of sequence structure with repetitive fragments. Two promoter regions containing polymorphic nucleotides were able to bind protein. We have detected differences that might guide future efforts to understand transcriptional differences of Pb*GP43 *among isolates.

## Methods

### Fungal isolates and growth conditions

*P. brasiliensis *clinical isolates Pb18, Pb3 (originally 608) and Pb339 (B-339) were the focus of this work. Genetic material from Pb2 (originally 1925), Pb4 (originally 1014), Pb5 (originally AP), Pb9 (originally 924), Pb10 (originally Peru), Pb11 (originally Mg5), Pb12 (originally Argentina), Pb14 (originally 470), Pb16 (originally solo) and Pb17 (originally tatu) were also analyzed for polymorphism in the 3' UTR and poly(A) cleavage site and/or length polymorphism of the 5' intergenic region. Details about the origin of these isolates, as well as their genetic groups according with the Pb*GP43 *phylogeny and multilocus studies can be found elsewhere [3,15]. The isolates were maintained in the yeast phase in slants of modified YPD medium (mYPD, 0.5% yeast extract, 0.5% casein peptone, 1.5% glucose, pH 6.3) either at 36°C, with sub-culturing every 20 days, or at 4°C for some months.

### Extraction of total DNA, RNA and preparation of total protein extracts

Total protein extracts used in DNA binding assays were obtained as detailed previously [23], using *P. brasiliensis *yeast cells from Pb18, Pb3 and Pb339 incubated at 36°C in mYPD with shaking (120 r.p.m.) for four to five days. Total DNA-free RNA was isolated from Pb18, Pb3 and Pb339 yeast cells using approximately 0.1 ml of wet pellets and the TRizol reagent (Invitrogen), as previously described [22]. For RNA extraction followed by real time RT-PCR, fungal cells were cultivated at 36°C with shaking in F12 medium (Gibco) supplemented with 1.5% glucose (F12/glc). Transcription modulation with fetal bovine serum (FBS) was verified by stimulating log-phase yeast cells growing in F12/glc with 2% FBS for 30 min. For transcription modulation with glucose, log-phase cultures in F12 medium (that has 0.18% glucose in its formulation) were supplemented with glucose to 1.5% final concentration. Total RNA-free DNA was purified from mechanically disrupted *P. brasiliensis *yeast cells cultivated in mYPD [12,15].

### Electrophoretic mobility shift assays (EMSA)

We followed EMSA protocols described by Tosco et al. [37] with annealed sense (Table 1) and anti-sense oligonucleotides, as detailed in our previous reports [22,23]. Briefly, double-stranded oligonucleotides (60,000 c.p.m) were radio labeled with [γ^32^P] dATP (10 mCi/ml, Amersham) and incubated (15 min at 37°C) with an ice-cold solution containing 10 μg of total protein extract from *P. brasiliensis*, 1.5 μL of poly dI-dC (1.25 mg/mL), 1.5 μL of BSA (10 mg/mL) and 3 μL of a solution containing 125 mM Hepes, pH 7.5, 5 mM EDTA and 50% glycerol in a total reaction volume of 12 μl. Competition assays were incubated in the presence of molar excess of cold oligonucleotides. The reactions were separated in 6% non-denaturing polyacrylamide gels (37.5:1 acrilamide/bis-acrilamide) run in 0.5 × TBE buffer either for 45 min at 100 V in a mini-Protean II apparatus (BioRad), or for one hour at 20 mA in 14 × 12 cm gels. The gels were dried and exposed to an X-Omat (Kodak) film at -70° C.

### Cloning an extended fragment of the 5' intergenic region of PbGP43

We developed a strategy to clone an extended fragment of the Pb*GP43 *5' intergenic region using PCR and a combination of primers from i) an internal 5' region of the Pb*GP43 *ORF (PCRia, reverse primer, 5'-GCGAGAACACAGCTGGCAAGAGCCAGGTTAAGAG-3'); ii) conserved ORF regions from the 5' neighboring gene of fungal β-1,3-glucanases homologous to Pb*GP43 *(forward consensus primers). By the time we used that strategy there was publicly available genome information from *Aspergillus fumigatus *http://www.tigr.org/tdb/e2k1/afu1/, *A. nidulans *and *A. terreus *http://www.broad.mit.edu/node/568. We also counted on *H. capsulatum *and *B. dermatitidis *preliminary sequencing data kindly supplied by Dr. William E. Goldman, presently at the Duke University Medical Center. We found in *H. capsulatum*, *A. terreus *and *A. nidulans *a homologous GPI-anchored protein ORF lying 5.5 kb to 9.2 kb away from the β-1,3-glucanase gene. Three primers were designed from homologous DNA internal regions from that ORF. A series of PCR reactions were carried out at different annealing temperatures and primer combinations using a Long PCR Enzyme kit (Fermentas). Primers were also tested individually to control for unspecific bands. The PCR reactions were visualized in ethidium bromide gels, then Southern-blotted and hybridized with a probe covering 110 bp of the Pb*GP43 *5' proximal flanking region. A 1.8-kb fragment hybridized more strongly than others with the radioactive probe, and although it was the product of PCRia primer alone, it was cloned in pGEM-T vector and sequenced. Sequence information and a series of subsequent PCR, using selected primers from the newly sequenced region paired with ORF primers, showed that we managed to fortuitously clone an extended part of the 5' intergenic region to a total of 2,047 bp (updated U26160.2). For subsequent length polymorphism studies of this region, we compared amplicons obtained with internal Pb*GP43 *reverse primer (GRN, 5'-GAGGATCCCATGATGCCTATGCC-3') and forward P4 primer (5'-CAGCAGCATATTTGATTTCCT-3'), as shown in Results.

### 3' RACE RT-PCR

We used 3' RACE RT-PCR to obtain individual Pb*GP43 *transcripts and further compare their sequences and poly(A) sites. The reactions were assayed using the ThermoScript RT-PCR System (Gibco) and total DNA-free RNA from 10 *P. brasiliensis *isolates. Total cDNA was elongated using a standard oligo-dT primer (5'-GACTCGAGTCGACATCGT_17_-3'). The second strand and DNA amplifications were obtained with a forward Pb*GP43 *internal primer located at the 3' end (5'-CGATGCTCGCTTCCTCAT-3') and reverse corresponding to oligo-dT without the T-tail (5'-GACTCGAGTCGACATCG-3'). PCR reactions (100 μL) were carried out in 50 mM KCl, 1.5 mM MgCl_2_, 10 mM Tris-HCl, pH 9.0, 50 μM of each dNTP, 1 μM of each primer and 5 U Taq polimerase (Amersham). Cycling involved 5 min at 95°C, followed by 30 cycles at 95°C (1 min), 55°C (1 min) and 72°C (3 min, then 10 min). The amplified products were cloned into a pGEM-T vector (Promega). A series of transformed bacterial clones were selected for plasmid purification and insert sequence analysis.

### Quantitative real time RT-PCR

Quantitative real time RT-PCR was carried out using the Syber Green detection system (Applied Biosystems), following the manufacturer's instructions and details provided in our previous report [22]. The Pb*GP43 *ORF primers used in the reactions were 5'-TCGTGATATAGACAGCACCGTTG-3' (forward) and 5'- AAGACTTGGTTGTGGTATGTGTCG-3' (reverse). *P. brasiliensis *α-tubulin gene was used as calibrator with primers 5'-CGGCTAATGGAAAATACATGGC-3' (forward) and 5'-GTCTTGGCCTTGAGAGATGCAA-3' (reverse). Cycling was performed in an ABI Prism 7000 Sequence Detection System (Perkin-Elmer Applied Biosystem, USA) starting with one cycle of 50°C (2 min) and 95°C (1 min), followed by 40 cycles at 95°C (15 sec) and 60°C (1 min), and an additional cycle of 95°C (15 sec), 60°C (20 sec) and 95°C (15 sec) for determination of the dissociation curve. Negative controls did not contain DNA or RNA. Reactions were run in triplicates and in parallel with the α-tubulin calibrator. We built a standard curve for each probe by assaying increasing amounts of theoretical copy numbers of each gene obtained with serial dilutions of *P. brasiliensis *genomic DNA, as described [38]. The final data were presented as the mean ± SD.

### Sequence analysis

Nucleotide sequencing was carried out in the facilities of the Center of Human Genome at the São Paulo University (USP). Manual sequencing of 3' RACE products was carried out as described [15]. Sequences were analyzed using the EditSeq, SeqMan and MegAlign programs of the Lasergene System (DNAstar Inc.). Putative transcription motifs were deduced by the TFSearch program http://www.cbrc.jp/research/db/TFSEARCH.html.

## Authors' contributions

AAR participated in the preparation of the manuscript, designed and performed EMSA experiments with the Et probes, cloned, assembled and analyzed the expanded 5' flanking region, performed RT-PCR experiments; FVM designed and performed EMSA experiments with Bs probes, sequenced and analyzed polymorphisms of the 3' flanking region; RP gained funds to develop the projects, wrote the manuscript, analyzed data and supervised the development of the Ph.D. projects from AAR and FVM, whose partial data are contained in this manuscript. All authors read and approved the final manuscript.
